# Kombucha Tea Fortified With *Spirulina Maxima* and *Haematococcus pluvialis*: Impact on Physicochemical Properties, Bioactive Profile, and Antioxidant Capacity

**DOI:** 10.1002/fsn3.72150

**Published:** 2026-07-21

**Authors:** Ayşe Janseli Denizkara, Gökhan Akarca

**Affiliations:** ^1^ Food Engineering Department Afyon Kocatepe University Afyonkarahisar Türkiye

**Keywords:** antioxidant activity, astaxanthin, functional fermented beverage, kombucha

## Abstract

This study investigated the effect of adding *Spirulina maxima* and *Haematococcus pluvialis* microalgae to kombucha tea. Both additives increased membrane weight in a dose‐dependent manner. pH decreased with *S. maxima* and increased with *H. pluvialis* . Both microalgae significantly improved the overall antioxidant capacity, total phenolic content, and textural properties (cohesiveness, consistency, firmness, and viscosity index) of kombucha samples. *S. maxima* produced superior physicochemical parameters and organic acid concentrations compared to *H. pluvialis* . Concentrations of acetic, lactic, gluconic, and D‐glucuronic acids were highest in the 4% *S. maxima* samples. Furthermore, *S. maxima* promoted the accumulation of several phenolic compounds, including chlorogenic, gallic, caffeic, vanillin, and ferulic acids, whereas *H. pluvialis* increased the concentrations of astaxanthin, salicylic acid, quercetin, and kaempferol. In contrast, the addition of both microalgae reduced some catechin derivatives. Consequently, the integration of *S. maxima* and *H. pluvialis* into kombucha enhanced its nutritional value, antioxidant capacity, and bioactive compound richness.

## Graphical abstract

The fortification of kombucha tea with microalgae significantly enhances its bioactive profile, where *Spirulina maxima* (4%) acts as the optimal fermentation enhancer by increasing phenolics and organic acids, while *Haematococcus pluvialis* (4%) serves as the best astaxanthin source, with both microalgae contributing to elevated antioxidant activity.

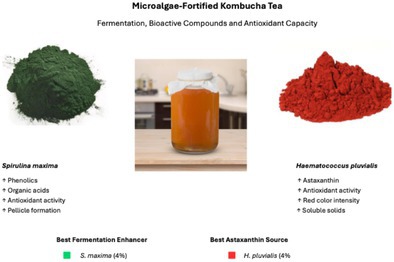

## 1 Introduction

Kombucha tea is a popular fermented beverage known for its unique taste and potential health benefits. This tea is typically prepared using sweetened tea (mostly black or green) and a symbiotic culture of bacteria and yeast (SCOBY). During the fermentation process, various bioactive compounds are produced that contribute to the beverage's characteristics and health benefits. Among the main components of kombucha are tea polyphenols, amino acids, vitamins, minerals, and various organic acids such as acetic acid, gluconic acid, and glucuronic acid. This beverage is rich in antioxidants, particularly attributed to the polyphenolic compounds released during fermentation (Batista et al. 2023).

The composition of kombucha can vary significantly depending on the tea base used (black, green, or herbal tea) and the fermentation time. Generally, longer fermentation leads to lower pH levels due to increased formation of organic acids (Martin‐Gómez et al. 2023). Antioxidant activity is significantly higher in fermented kombucha compared to unfermented tea, primarily due to microbial activities that alter the tea's polyphenol content (Aji et al. 2023).

*S. maxima* , a filamentous unicellular cyanobacterium belonging to the *Oscillatoraceae* family, has gained significant attention in recent years as a nutritionally rich food supplement. *S. maxima* , found primarily in alkaline water sources across Africa, Asia, and the Americas, stands out for its high protein content, essential amino acids, vitamins, minerals, and bioactive compounds that offer various health benefits. *Spirulina maxima*, with a protein content of 60%–71%, contains essential fatty acids, particularly gamma‐linolenic acid (GLA), numerous vitamins (especially B vitamins and vitamin E), and various minerals including iron (Gaur et al. 2024). In addition, it exhibits significant antioxidant activity, which plays an important role in reducing oxidative stress and preventing cellular damage. It has also been reported to contribute to the reduction of blood pressure and cholesterol levels (El‐Sheekh et al. 2014; Martínez‐Sámano et al. 2018).

*H. pluvialis* is a single‐celled green microalga belonging to the *Haematococcaceae* family. *H. pluvialis* , found in various freshwater habitats including rivers, lakes, and streams, is attracting attention for its nutritional and pharmaceutical potential (Sipaúba‐Tavares et al. 2023).

The primary characteristic of *H. pluvialis* is its high astaxanthin content, which is significantly higher than that found in other natural sources. Astaxanthin is described as a “super antioxidant” due to its ability to scavenge free radicals. This microalga also contains essential proteins, lipids, carbohydrates, amino acids, and various vitamins, which contribute to its high nutritional value. Additionally, due to its ability to accumulate various carotenoids, fatty acids, and polysaccharides, it is becoming an important component for functional foods (Nemani et al. 2024).

Research has shown that astaxanthin derived from *H. pluvialis* exhibits antioxidant effects that can help reduce oxidative stress, which plays a role in many chronic diseases, including cardiovascular disorders and neurodegenerative diseases (Song et al. 2022). It has also been reported that astaxanthin has effects that can alleviate conditions such as arthritis and improve skin health by providing protection against UV damage (Ha et al. 2024).

Although microalgae have been increasingly utilized in functional foods, specific comparative studies evaluating the distinct fermentation dynamics of kombucha fortified with astaxanthin‐rich *H. pluvialis* versus protein‐ and phenolic‐rich *S. maxima* remain extremely limited. This study addresses this current knowledge gap by providing a comprehensive comparison of how these two distinct microalgae impact the physicochemical properties, bioactive profile, and overall antioxidant capacity of kombucha tea. Consequently, this study aims to investigate the specific effects of adding *S. maxima* and *H. pluvialis* powders to kombucha at varying concentrations to elucidate their distinct roles in functional beverage formulation.

Recent studies highlight the growing trend of utilizing microalgae and novel substrates to enhance the functional and nutritional properties of various food products (Alkan et al. 2026). The integration of bioactive‐rich ingredients into complex food matrices has been shown to significantly improve antioxidant capacities and overall health benefits (Ganimet et al. 2026; Koh et al. 2026). Furthermore, recent advancements in extraction and fermentation technologies have allowed for a better preservation of these temperature‐sensitive bioactive compounds during processing (Rodrigues et al. 2026; Türkol et al. 2026). From a broader perspective, utilizing such functional ingredients not only boosts the biological profile of beverages like kombucha but also aligns with modern sustainable food system approaches and novel formulation strategies (Alkan et al. 2025; Salinas‐Ruiz et al. 2026).

## 2 Material and Methods

### 2.1 Production of Kombucha Teas

Kombucha teas were produced according to the parameters specified by Atik et al. (2025). In production, 25 g of black tea ( *Camellia sinensis* ) was weighed using a precision scale and 1000 mL of drinking water was added. 100 g/L of sucrose was added to the mixture, and after it was completely dissolved, it was heat‐treated at 95°C for 20 min. Then the mixture was left to steep for 20 min. The mixture was then filtered using sterile filter paper (Whatman No. 32). Subsequently, the mixture was placed in closed jars and sterilized in an autoclave (Nüve‐OT 90 L, Turkey) at 121°C and 1 atm pressure for 20 min. After sterilization, the mixture was cooled to 25°C. Subsequently, 2% (w/v) and 4% (w/v) mixtures of *H. pluvialis* and *S. maxima* powders were added. Finally, 45 g/L of pellicle phase and 150 mL of liquid phase from previously produced Kombucha teas were added to the samples, and all samples were left to ferment for 21 days in a dark environment at 24°C ± 3°C. A 21 day fermentation period was selected based on preliminary trials to allow sufficient time for the slow‐release bioactive compounds from the microalgae to effectively integrate into the beverage matrix. Furthermore, the SCOBY inoculum and liquid starter were exclusively sourced from the same active mother culture batch to ensure microbial consistency across all samples.

### 2.2 Cyanobacterium and Green Microalga Used in the Study

The *H. pluvialis* and *S. maxima* powders used in the study were obtained from Orzax (Istanbul, Türkiye) and Wefood (Istanbul, Türkiye), respectively. According to the manufacturers' specifications, the commercial *S. maxima* powder contained approximately 60% protein, while the *H. pluvialis* powder contained approximately 1.5% astaxanthin.

### 2.3 Physicochemical Analyses

The pH values of the kombucha tea samples were determined using a calibrated pH meter (Sartorius CP224S, Germany) according to Chen et al. (2025), and the soluble dry matter content (°Brix) values were determined using a desktop refractometer (Atago Rx 5000, Japan) according to Yıkmış and Tuğgüm (2019). The pellicle weights of the samples were measured using a precision balance (Radwag PS‐1000 R2, Poland). DPPH radical scavenging activity was determined according to Pavithra and Vadivukkarasi (2015), ABTS radical scavenging activity according to Re et al. (1999), and total phenolic content (TPC) according to Gulcin et al. (2002) using gallic acid as the calibration standard. The TPC values were expressed as mg gallic acid equivalents per 100 mL of kombucha sample (mg GAE/100 mL). The color values (L*, a*, and b*) of the kombucha tea samples were determined using a Hunter colorimeter (Konica Minolta Chroma Meter CR‐400) according to Abuduaibifu and Tamer (2019).

### 2.4 Texture Analysis

The texture parameters of the tea samples (hardness, consistency, cohesiveness, and viscosity index) were determined using a TA.XT Plus texture analysis instrument (Stable Micro Systems, Godalming, Surrey, UK) according to the method described by Akarca and Denizkara (2024) using the back extrusion technique. For this purpose, the pressing process was performed at a crosshead speed of 1 mm.s‐1 by immersing a cylindrical acrylic back extrusion probe with a diameter of 7.62 mm and a depth of 10 mm into the tea samples at 25°C. Power‐time graphs were generated for each probe immersion, based on positive areas when the probe was inserted and negative areas when it was removed. Texture Exponent 32 (2007) software (Stable Micro Systems, Godalming, United Kingdom) was used to determine the textural properties of the samples. The evaluated parameters were determined as hardness (g) maximum positive force, consistency (gs) positive region area, cohesion maximum negative force, and viscosity index (gs) negative region area.

### 2.5 Microbiological Analyses

Microbiological analyses were initiated by preparing serial dilutions. 10 mL was taken from each sample and 90 mL of sterile Ringer's solution prepared at ¼ strength with Ringer's tablets (Merck Millipore, 1,155,251, Germany) was added, and the mixture was homogenized in a stomacher (Bagmixer 400P). Later, other decimal dilutions were prepared using this obtained dilution. Microbiological analyses were performed on these serial dilutions using the spread plate technique. Analysis of lactic acid bacteria count in the samples was performed using Man Rogosa and Sharpe (MRS) Agar (Merck 1.10661, Germany) under anaerobic conditions at 30°C for 24–48 h. Acetic acid bacteria count analysis was performed using Yeast Extract Calcium Carbonate Glucose (YCG) Agar (Himedia M1182, India) under aerobic conditions at 30°C for 5–10 days (Neffe‐Skocińska et al. 2017). *Lactococcus/Streptococcus* species bacteria count analysis was performed using M‐17 Agar (Merck, 1.15108, Germany) under aerobic conditions at 30°C for 24–48 h (Öner et al. 2010).

### 2.6 Organic Acid Analyses

The organic acid concentrations of the samples were determined using an HPLC (Shimadzu Prominence, Shimadzu Corporation, Kyoto, Japan) instrument. For analysis, 4 g of the sample was weighed, 20 mL of 0.01 N H2SO4 was added, and the mixture was vortexed. It was then fed into the system using a 0.45 μm filter (Güzel‐Seydim et al. 2000). System characteristics used: CBM: 20ACBM; detector: DAD (SPD‐M20A); column oven: CTO‐10ASVp; pump: LC20 AT; autosampler: SIL 20ACHT; computer program: LC solution; column: ODS 4 (250 mm × 4.6 mm, 5 μm; Inertsil ODS‐4, GP Sciences, Nakatsugawa, Japan); mobile phase: ultra‐pure water adjusted to pH 3 with orthophosphoric acid. The mobile phase flow rate was 0.7 mL min‐1 and detection was performed at a wavelength of 210 nm (Hakan et al. 2005).

Identification and quantification of organic acids were performed by comparing the retention times and peak areas with those of authentic external standards (Sigma‐Aldrich, USA). The calibration curves exhibited high linearity with correlation coefficients (*R* ^2^) ≥ 0.99. Limits of detection (LOD) and limits of quantification (LOQ) were established based on standard signal‐to‐noise ratios of 3:1 and 10:1, respectively.

### 2.7 Phenolic Composition

The phenolic composition of kombucha samples was determined using high‐performance liquid chromatography (HPLC). Filtered samples (2 mL) were passed through a membrane filter (0.45 μm) and transferred to HPLC vials. A 10 μL aliquot of the obtained filtrate was separated using a reversed‐phase column (Mightysil RP‐18 GP 250 mm × 4.6 mm, 5 μm; Kanto Corporation, Portland, OR, USA) (Nuutila et al. 2002). The HPLC system (Hitachi) is equipped with an autosampler (L‐2200; Hitachi) and a photodiode array detector (L‐2455; Hitachi). The injection volume was 10 μL, and a gradient was applied using pump (L‐2130; Hitachi), solvent A (containing 0.1% phosphoric acid, 0.1% acetonitrile, and 5% N,N‐dimethylformamide) at a flow rate of 0.8 mL/min and solvent B (100% acetonitrile). The gradient illusion started at 100% A (0% B solvent) and linearly increased to 100% B at the end of a 50 min cycle. The analyses were monitored at a wavelength of 280 nm.

Phenolic compounds were identified by matching their retention times and UV–Vis spectra with external standard compounds (Sigma‐Aldrich, USA). Quantification was achieved using multi‐point calibration curves (*R* ^2^ ≥ 0.99). The analytical validation parameters (LOD, LOQ, and recovery) fell within acceptable limits for routine food matrix analysis.

### 2.8 Statistical Analyses

The experimental design consisted of two independent fermentation batches (biological replicates). All physicochemical, textural, and bioactive assays were performed in duplicate (analytical replicates) for each independent batch. In this study, a two‐way analysis of variance (ANOVA) was used to evaluate the differences between the samples, considering the type of microalgae and the concentration as factors. The analysis findings were subjected to Duncan's multiple range tests to determine the statistical differences among the means at a significance level of *p* < 0.05 using SPSS software. For ease of presentation in tables, the samples were coded as follows: Control (no microalgae), R1 (2% *H. pluvialis* ), R2 (4% *H. pluvialis* ), G1 (2% *S. maxima* ), and G2 (4% *S. maxima* ).

## 3 Result and Discussion

### 3.1 pH, Brix, and Pellicle Weight

Compared to the control sample, the addition of *S. maxima* to kombucha tea samples caused a decrease in pH values, while the addition of *H. pluvialis* led to an increase. Additionally, the rate of change in pH values also increased depending on the amount of *Spirulina maxima* and *Haematococcus pluvialis* added (Table 1; *p* < 0.05).

**TABLE 1:** pH, % brix and pellicle weight values of kombucha samples.

Samples	pH	Brix (%)	Pellicle weight (g)
Control	1.93 ± 0.01^c^	6.69 ± 0.02^d^	73.44 ± 3.13^c^
R1	2.05 ± 0.02^b^	8.90 ± 0.02^a^	75.22 ± 1.44^bc^
R2	2.10 ± 0.02^a^	8.97 ± 0.02^a^	78.22 ± 1.22^abc^
G1	1.90 ± 0.01^cd^	6.38 ± 0.05^c^	79.23 ± 1.35^ab^
G2	1.87 ± 0.01^c^	6.21 ± 0.04^b^	82.64 ± 2.02^a^
Interactions	*p*		
Samples (S)	< 0.001	< 0.001	0.029
Concentration (C)	0.022	0.004	0.069
S X C	0.451	0.077	0.890

*Note:* R1: 2% *Haematococcus pluvialis* , R2: 4% *Haematococcus pluvialis* , G1: 2% *Spirulina maxima*, G2: 4% *Spirulina maxima*, a–d (↓): Values with the differ lowercase letters in the same column for each analysis differ significantly (*p* < 0.05) Statistical significance: *p* < 0.001: highly significant; *p* < 0.01: very significant; *p* < 0.05: significant.

Among the samples, the lowest brix (%) value was found in the sample produced with 4% *S. maxima* , with a value of 6.21%, while the highest brix value was found in the sample produced with 4% *H. pluvialis* , with a value of 8.97% (Table 1). Compared to the control sample, the brix degree of samples with *S. maxima* added was lower, while the brix degrees of samples with *H. pluvialis* added were found to be higher (*p* < 0.05).

The pellicle weights of the kombucha samples increased with the addition of *H. pluvialis* and *S. maxima*, and in parallel with the amount added (*p* < 0.05). Among the samples, the highest pellicle weight was found in the samples produced with the addition of 4% *S. maxima* , with 82.64 g. The sample was followed by tea samples produced with the addition of 79.23 g and 2% *S. maxima*, and 78.22 g and 4% *H. pluvialis* , respectively (Table 1).

*S. maxima* contains various bioactive compounds, including phenols and flavonoids. These compounds serve as a substrate for fermentative microorganisms, increasing organic acid production and accelerating the pH drop resulting from fermentation. Additionally, it has led to a decrease in brix values by allowing microorganisms to metabolize the sugars in the environment more. Consequently, as microbial growth was greater, the pellicle weights of these samples also showed a more significant increase compared to the control sample (Mahmoud et al. 2022). In contrast, astaxanthin, a carotenoid produced by *H. pluvialis* , affected microbial growth rates and metabolic efficiency responsible for fermentation, ultimately slowing down organic acid production (Jannel et al. 2020). This situation has caused the pH levels to remain high compared to the control sample. Additionally, because microbial metabolism develops more slowly, the brix values remained high and pellicle development slowed.

It should be noted that the final pH values (1.87–2.10) are substantially lower than the typical kombucha pH range (2.5–3.5). The final pH values (1.87–2.10) are substantially lower than the typical kombucha pH range (2.5–3.5). The unusually low pH values observed in this study require further investigation. Although the extended fermentation period and the supplementation with microalgae may have contributed to increased acidification, the present study did not include titratable acidity measurements or fermentation kinetic monitoring. Therefore, the factors responsible for the observed pH reduction cannot be conclusively identified, and additional studies incorporating titratable acidity analyses and fermentation kinetics are required to confirm the underlying mechanisms.

### 3.2 DPPH (2,2‐Diphenyl‐1‐Picrylhydrazyl), ABTS (2,2′‐Azino‐Bis (3 Ethylbenzothiazoline‐6‐Sulfonic Acid), and Total Phenolic Content (TPC) Values

The DPPH, ABTS, and TPC values of the samples increased in parallel with the addition of *H. pluvialis* and *S. maxima* (*p* < 0.05). The highest DPPH, ABTS, and TPC values were found in samples produced with 4% *S. maxima* addition, with values of 92.62%, 8.48 μmol TE/g, and 305.83 mg GAE/mL, respectively. Conversely, the lowest DPPH, ABTS, and TPC values were observed in control samples, with values of 53.29%, 7.16 μmol TE/g, and 183.69 mg GAE/100 mL, respectively. Additionally, it was determined that the addition of *S. maxima* increased the DPPH, ABTS, and TPC values of the samples more than the addition of *H. pluvialis* (Table 2).

**TABLE 2:** % DPPH, ABTS and TPC values of kombucha samples.

Samples	DPPH (%)	ABTS (μmol TE/g)	TPC (mg GAE/100 mL)
Control	53.29 ± 2.73^d^	7.16 ± 0.11^d^	183.69 ± 3.45^d^
R1	69.49 ± 1.49^c^	7.95 ± 0.05^c^	258.78 ± 3.48^c^
R2	78.73 ± 3.40^b^	8.11 ± 0.03^bc^	272.40 ± 5.28^b^
G1	84.65 ± 1.99^b^	8.20 ± 0.06^b^	279.84 ± 7.32^b^
G2	92.62 ± 2.17^a^	8.48 ± 0.04^a^	305.83 ± 7.89^a^
Interactions	*p*		
Samples (S)	< 0.001	0.001	0.004
Concentration (C)	0.004	0.005	0.001
S X C	0.728	0.231	0.851

*Note:* R1: 2% *Haematococcus pluvialis* , R2: 4% *Haematococcus pluvialis* , G1: 2% *Spirulina maxima*, G2: 4% *Spirulina maxima*, a–d (↓): Values with the differ lowercase letters in the same column for each analysis differ significantly (*p* < 0.05) Statistical significance: *p* < 0.001: highly significant; *p* < 0.01: very significant; *p* < 0.05: significant.

The addition of *S. maxima* and *H. pluvialis* not only introduces their natural antioxidants to the fermentation medium but also enhances the overall bioactive profile of kombucha tea. The high antioxidant mechanisms of these two microalgae lead to a significant increase in kombucha's DPPH and ABTS activities by neutralizing reactive free radicals in the environment. In this way, both the antioxidant activity of the beverage and an increase in its sensory properties are observed (Bassani et al. 2025). The synergistic effects between fermentation agents and microalgae create a transformative environment where phenolic compound interaction occurs. In fermentations with the addition of phenolic‐rich algae such as *S. maxima* and *H. pluvialis* , during fermentation, the microbial enzymes catalyze the *depolymerization* and hydrolysis of complex polyphenols into simpler, more highly reactive phenolic acids. This structural breakdown exposes more hydroxyl groups, which explains the significant increase in TPC values observed at the end of fermentation (Doğan and Doğan 2023).

### 3.3 Color Values

**FIGURE 1:**
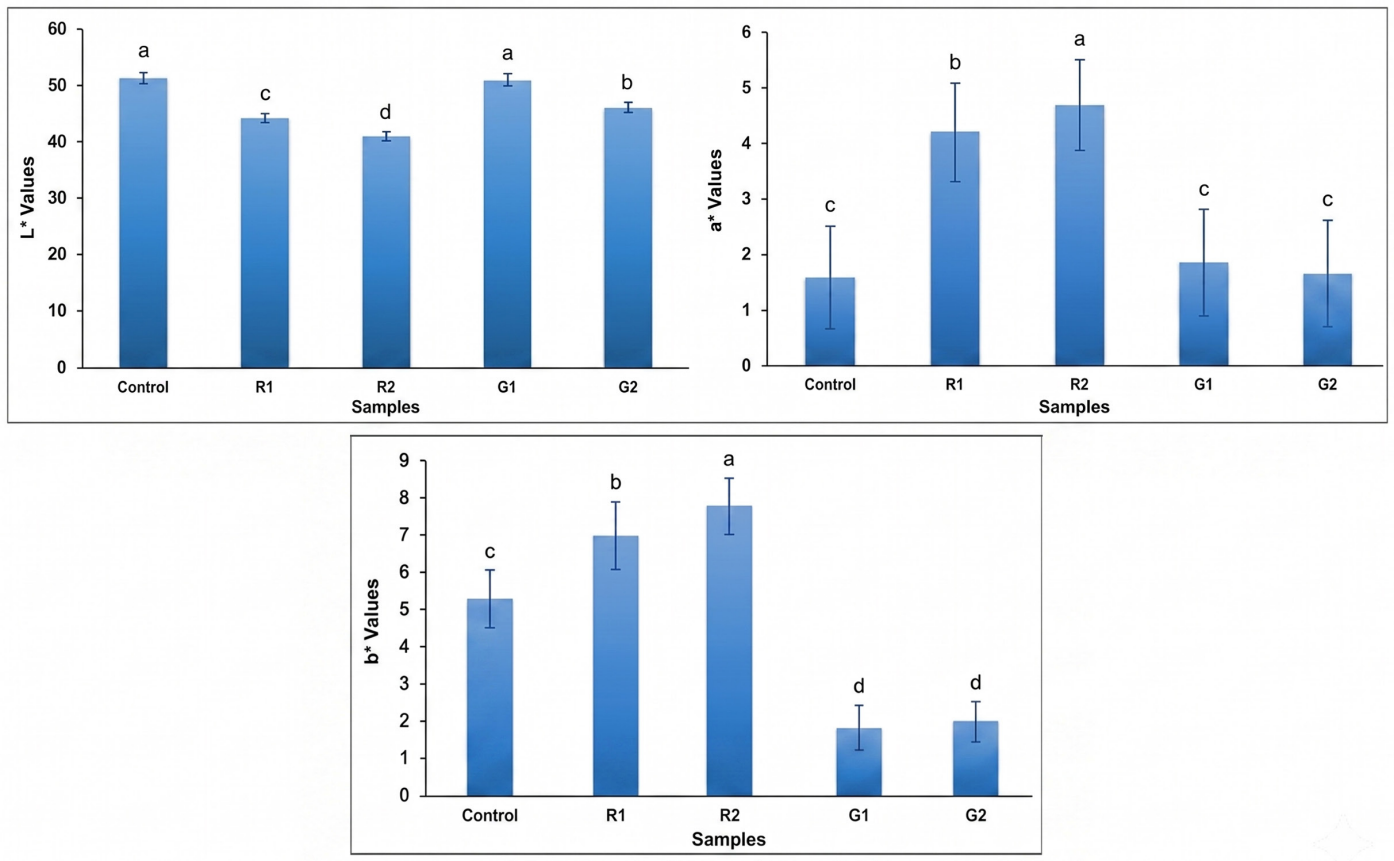
The L*, a*, and b* values of kombucha samples (a). Abbreviations are: R1: 2% *Haematococcus pluvialis* , R2: 4% *Haematococcus pluvialis* , G1: 2% *Spirulina maxima*, G2: 4% *Spirulina maxima*.

The addition of *S. maxima* and *H. pluvialis* showed significant changes in the L*, a*, and b* values of the tea samples (*p* < 0.05). Although the addition of both microalgae reduced the L* value compared to the control sample, this reduction was less pronounced in the samples with added *S. maxima* . The a* values of the samples were particularly higher in products with the addition of *H. pluvialis* (4.14 and 4.74) due to astaxanthin, a strong carotenoid pigment they contain. b* values, similarly, reached their highest values (7.04 and 7.85) in samples with the addition of *H. pluvialis* . In contrast, the samples produced with the addition of *S. maxima* were found to be lower (1.83 and 1.98) than the control samples (Figure 1).

When chlorophyll levels decrease during fermentation, there can be a relative increase in the visibility of theaflavins and thearubigins originating from the black tea base. The addition of *S. maxima* particularly promotes the breakdown of chlorophyll during the fermentation process while interacting with tea‐derived pigments, which affects the perception of the color yellow (Ren et al. 2019). This situation can affect the perception of the color yellow (Mattioli et al. 2020).

### 3.4 Textural Values

Although kombucha is a liquid beverage, measuring its rheological and textural properties (such as consistency and viscosity) is crucial in this context. The addition of microalgal proteins and exopolysaccharides alters the mouthfeel and structural stability of the beverage, which are critical parameters for consumer acceptance of novel functional drinks.

The addition of *S. maxima* and *H. pluvialis* to kombucha tea samples caused an increase in all textural values (cohesiveness, consistency, firmness, and viscosity index) of the samples (*p* < 0.05). The increase in the amount of algae added has also increased in proportion to the amount added. The addition of *S. maxima* from two different microalgae increased textural values more than *H. pluvialis* . Among the samples, the highest values for cohesiveness (−7.07 g), consistency (139.79 g.s), firmness (12.62 g), and viscosity index (−0.43 g.s) were found in the samples produced with 4% *S. maxima* addition. These samples were followed by those produced with 2% *S. maxima* and 4% *H. pluvialis* addition (Table 3).

**TABLE 3:** Textural analysis values of kombucha samples.

Samples	Cohesiveness (g)	Consistency (g.s)	Firmness (g)	İndex of viscosity (g.s)
Control	−9.66 ± 0.16^c^	101.67 ± 2.34^c^	11.67 ± 0.02^d^	−0.60 ± 0.04^b^
R1	−8.90 ± 0.33^c^	114.14 ± 4.16^c^	11.76 ± 0.03^d^	−0.70 ± 0.10^b^
R2	−8.72 ± 1.02^bc^	121.01 ± 6.54^b^	12.00 ± 0.06^c^	−0.87 ± 0.07^c^
G1	−7.48 ± 0.10^ab^	129.53 ± 1.64^b^	12.23 ± 0.04^b^	−0.59 ± 0.03^b^
G2	−7.07 ± 0.34^a^	139.79 ± 3.24^a^	12.62 ± 0.06^a^	−0.43 ± 0.03^a^
Interactions	*p*			
Samples (S)	0.003	< 0.001	< 0.001	0.029
Concentration (C)	0.168	0.003	< 0.001	0.019
S X C	0.644	0.163	0.076	0.474

*Note:* R1: 2% *Haematococcus pluvialis* , R2: 4% *Haematococcus pluvialis* , G1: 2% *Spirulina maxima*, G2: 4% *Spirulina maxima*, a—d (↓): Values with the differ lowercase letters in the same column for each analysis differ significantly (*p* < 0.05) Statistical significance: *p* < 0.001: highly significant; *p* < 0.01: very significant; *p* < 0.05: significant.

The addition of microalgae such as *S. maxima* and *H. pluvialis* improves textural values due to their protein compositions. Proteins obtained from *S. maxima* and *H. pluvialis* play a critical role by forming a network that imparts firmness and reduces water activity during fermentation. This has led to an increase in cohesiveness and consistency values, resulting in a denser and more stable kombucha product due to the proteins interacting with the other components of brewing and contributing to the overall texture. The addition of these microalgae has shown a significant impact on the harshness and viscosity index of kombucha tea. The structural integrity provided by the cell wall composition of *S. maxima* and *H. pluvialis* contributed to the firmness and viscosity index properties of the tea (Ramalho et al. 2022). Furthermore, the fermentation process alters the phenolic content and antioxidant capacity of kombucha, which is significant not only in terms of flavor and health benefits but also contributes to the product's overall texture. Increased levels of these compounds lead to a darker beverage and further enhance the sensory experience. Additionally, polysaccharides such as β‐glucans and exopolysaccharides produced during the fermentation of *S. maxima* and *H. pluvialis* can contribute to their increase, ultimately leading to an increase in viscosity (Jayabalan et al. 2014).

### 3.5 Microbiological Count

In kombucha samples, the counts of lactic acid bacteria (LAB), *Streptococcus/Lactococcus* (SLB), and acetic acid bacteria (AAB) were found to be higher in samples produced with the addition of *S. maxima* compared to the control sample, while they were lower in samples produced with the addition of *H. pluvialis* (*p* < 0.05). The highest lactic acid, *Streptococcus/Lactococcus*, and acetic acid bacterial counts were found in samples produced with 4% *S. maxima* addition, with values of 4.32, 4.24, and 5.38 log cfu/mL, respectively. Conversely, the lowest counts were found in samples produced with 4% *H. pluvialis* addition, with values of 4.03, 4.06, and 5.07 log cfu/mL, respectively (Table 4).

**TABLE 4:** Microbiological count of kombucha samples (log cfu/mL).

Samples	Lactic acid bacteria	*Lactococcus/streptococcus* species bacteria	Acetic acid bacteria
Control	4.14 ± 0.03^b^	3.97 ± 0.02^d^	5.20 ± 0.03^bc^
R1	4.11 ± 0.03^b^	4.10 ± 0.01^bc^	5.14 ± 0.01^c^
R2	4.03 ± 0.03^c^	4.06 ± 0.03^c^	5.07 ± 0.01^d^
G1	4.26 ± 0.04^a^	4.13 ± 0.02^b^	5.24 ± 0.03^b^
G2	4.32 ± 0.03^a^	4.24 ± 0.03^a^	5.38 ± 0.03^a^
Interactions	*p*		
Samples (S)	< 0.001	0.002	< 0.001
Concentration (C)	0.017	0.007	0.002
S X C	0.667	0.116	0.138

*Note:* R1: 2% *Haematococcus pluvialis* , R2: 4% *Haematococcus pluvialis* , G1: 2% *Spirulina maxima*, G2: 4% *Spirulina maxima*, a—d (↓): Values with the differ lowercase letters in the same column for each analysis differ significantly (*p* < 0.05) Statistical significance: *p* < 0.001: highly significant; *p* < 0.01: very significant; *p* < 0.05: significant.

In kombucha production, the addition of different substrates increases the biological activities of the tea (Villarreal‐Soto et al. 2018). *Spirulina* supplementation supports these dynamics through both direct and indirect mechanisms. *S. maxima* not only provided the necessary nutrients for microbial growth but also supported the structure of the microbial community, thereby increasing the proliferation of LAB, SLB, and AAB. In contrast, *H. pluvialis* has a high astaxanthin content. These bioactive compounds, which possess antimicrobial properties, can inhibit the growth of certain bacteria, particularly those sensitive to them, such as AAB and LAB, during the fermentation process (Zhang et al. 2024). Additionally, the addition of *H. pluvialis* leads to changes in the physicochemical environment of kombucha, such as its antioxidant level. High levels of antioxidants due to astaxanthin alter the oxidative state of the fermentation medium, making it less favorable for the growth of LAB and AAB (Hananta et al. 2025).

While the observed differences in bacterial counts between the samples are relatively small (< 0.3 log units), they indicate modest but significant shifts in the microbial consortium driven by the microalgal substrates. Furthermore, it is important to acknowledge that yeast populations, which are fundamental drivers of kombucha fermentation, were not quantified in this study. The lack of yeast population dynamics represents a limitation of the current work and should be comprehensively addressed in future studies.

### 3.6 Organic Acid Values

While the addition of *S. maxima* to kombucha production increased the organic acid values of the samples, the addition of *H. pluvialis* decreased them (*p <* 0.05). Among the samples, the highest acetic (4.98 g/L), lactic (0.39 g/L), gluconic (2.85 g/L), and D‐glucuronic acid (1.58 g/L) values were found in the samples produced with 4% *S. maxima* addition, while the lowest values were observed in the samples produced with 4% *H. pluvialis* addition (Table 5).

**TABLE 5:** Organic acid values of kombucha samples (g/L).

Samples	Acetic acid	Lactic acid	Gluconic acid	D‐Glucuronic acid
Control	4.74 ± 0.01^c^	0.23 ± 0.03^ab^	2.57 ± 0.05^c^	1.29 ± 0.05^cd^
R1	4.71 ± 0.02^cd^	0.21 ± 0.03^bc^	2.70 ± 0.03^b^	1.40 ± 0.05^bc^
R2	4.66 ± 0.03^d^	0.15 ± 0.01^c^	2.63 ± 0.03^bc^	1.28 ± 0.01^d^
G1	4.83 ± 0.03^b^	0.29 ± 0.04^a^	2.70 ± 0.03^b^	1.42 ± 0.04^b^
G2	4.98 ± 0.04^a^	0.39 ± 0.03^a^	2.85 ± 0.04^a^	1.58 ± 0.05^a^
Interactions	*p*			
Samples (S)	< 0.001	< 0.001	0.006	0.004
Concentration (C)	0.004	0.011	0.006	0.006
S X C	0.065	0.413	0.146	0.411

*Note:* R1: 2% *Haematococcus pluvialis* , R2: 4% *Haematococcus pluvialis* , G1: 2% *Spirulina maxima*, G2: 4% *Spirulina maxima*, a—d (↓): Values with the differ lowercase letters in the same column for each analysis differ significantly (*p* < 0.05) Statistical significance: *p* < 0.001: highly significant; *p* < 0.01: very significant; *p* < 0.05: significant.

It has been determined that the addition of *S. maxima* can enrich the microbial community involved in kombucha fermentation and increase the metabolic activity of specific microorganisms that produce organic acids (Phung et al. 2023). Its presence provides additional nutrients that support the development of AAB and LAB, which are necessary for organic acid production (Harrison and Curtin 2021). Additionally, *Spirulina* is composed of complex polysaccharides that can lead to the production of acids like lactic and acetic acid when broken down during fermentation. The complex structure of *Spirulina*, particularly its cell wall composed of polysaccharides, supports long‐term fermentation and increased organic acid yield by providing a slow‐release nutrient source. As fermentation progresses, the breakdown of *Spirulina*'s structural components contributes to the increased production of these acids during the metabolic activities of the SCOBY (Nguyen et al. 2015; Shioji et al. 2021).

Antioxidants scavenge free radicals and other reactive species that may be involved in the complex metabolic pathways during fermentation. We hypothesize that the specific bioactive components in *H. pluvialis* , such as astaxanthin, may have contributed to a less favorable environment for rapid acidogenic metabolism, which correlates with the lower organic acid concentrations observed at the endpoint. The presence and dynamics of specific bacterial and yeast species are crucial for acid production in kombucha. The addition of *H. pluvialis* could affect these microbial community dynamics and promote selective pressures that favor microorganisms producing less acidic metabolites (Shah et al. 2016).

### 3.7 Phenolic Compounds

Compared to control samples, the addition of Spirulina to kombucha increased the concentrations of chlorogenic, gallic, caffeic, vanillin, coumaric, ferulic, and cinnamic acids. In contrast, the addition of *H. pluvialis* led to an increase in the values of astaxanthin, salicylic acid, quercetin, and kaempferol. Additionally, the addition of microalgae caused a decrease in the values of catechin, gallocatechin, epicatechin, and epigallocatechin (*p* < 0.05; Table 6).

**TABLE 6:** Phenolic compounds of kombucha samples (mg/100 g).

Samples	Catechin	Gallocatechin	Epicatechin	Epigallocatechin
Control	20.70 ± 0.06^a^	13.84 ± 0.28^a^	1.36 ± 0.04^a^	2.43 ± 0.02^a^
R1	13.78 ± 0.06^d^	10.02 ± 0.08^cd^	1.24 ± 0.01^c^	2.09 ± 0.02^c^
R2	11.22 ± 0.14^e^	9.77 ± 0.16^d^	1.21 ± 0.01^c^	2.04 ± 0.02^d^
G1	18.31 ± 0.18^b^	11.37 ± 0.15^b^	1.31 ± 0.01^ab^	2.29 ± 0.01^b^
G2	16.36 ± 0.21^c^	10.49 ± 0.22^c^	1.26 ± 0.02^bc^	2.26 ± 0.01^b^
Interactions	*p*			
Samples (S)	< 0.001	< 0.001	0.056	0.004
Concentration (C)	< 0.001	0.008	0.014	0.006
S X C	0.032	0.068	0.562	0.411
Samples	Astaxanthin	Salicylic acid	Gallic Acid	Caffeic Acid
Control	0 ± 0.00^c^	0 ± 0.00^d^	6.34 ± 0.03^e^	0 ± 0.00^e^
R1	18.13 ± 0.06^b^	0.25 ± 0.04^bc^	8.60 ± 0.28^d^	1.33 ± 0.07^d^
R2	22.87 ± 0.19^a^	0.43 ± 0.04^a^	9.86 ± 0.11^c^	1.55 ± 0.03^c^
G1	0 ± 0.00^c^	0.10 ± 0.11^cd^	11.50 ± 0.42^b^	2.20 ± 0.03^b^
G2	0 ± 0.00^c^	0.33 ± 0.03^ab^	16.45 ± 0.45^a^	2.51 ± 0.04^a^
Interactions	*p*			
Samples (S)	< 0.001	0.029	< 0.001	< 0.001
Concentration (C)	< 0.001	0.004	< 0.001	< 0.001
S X C	< 0.001	0.581	< 0.001	0.151

Samples	Vanillin acid	Coumaric acid	Syringic acid	Chlorogenic acid
Control	0 ± 0.00^e^	0 ± 0.00^e^	0 ± 0.00^d^	0.78 ± 0.05^d^
R1	0.7 ± 0.09^d^	0.19 ± 0.02^d^	0.57 ± 0.05^b^	1.03 ± 0.03^c^
R2	1.01 ± 0.05^c^	0.37 ± 0.02^c^	0.67 ± 0.02^a^	0.91 ± 0.04^cd^
G1	1.19 ± 0.03^b^	0.46 ± 0.03^b^	0.36 ± 0.03^c^	1.91 ± 0.05^b^
G2	1.39 ± 0.08^a^	0.55 ± 0.03^a^	0.43 ± 0.03^c^	3.60 ± 0.08^a^
Interactions	*p*			
Samples (S)	< 0.001	< 0.001	< 0.001	< 0.001
Concentration (C)	0.002	< 0.001	0.010	< 0.001
S X C	0.296	0.003	0.511	< 0.001
Samples	Ferulic acid	Cinnamic acid	Quercetin	Kaempferol
Control	0 ± 0.00^e^	0 ± 0.00^e^	0 ± 0.00^e^	0 ± 0.00^e^
R1	2.77 ± 0.05^d^	10.77 ± 0.16^d^	2.92 ± 0.06^b^	4.67 ± 0.17^b^
R2	3.37 ± 0.06^c^	12.13 ± 0.06^c^	3.65 ± 0.05^a^	5.28 ± 0.08^a^
G1	4.47 ± 0.09^b^	14.25 ± 0.07^b^	0.66 ± 0.15^d^	1.89 ± 0.22^d^
G2	6.21 ± 0.04^a^	16.74 ± 0.16^a^	1.08 ± 0.05^c^	2.28 ± 0.11^c^
Interactions	*p*			
Samples (S)	< 0.001	< 0.001	< 0.001	< 0.001
Concentration (C)	< 0.001	< 0.001	< 0.001	0.004
S X C	< 0.001	0.001	0.049	0.314

*Note:* R1: 2% *Haematococcus pluvialis* , R2: 4% *Haematococcus pluvialis* , G1: 2% *Spirulina maxima*, G2: 4% *Spirulina maxima*, a—e (↓): Values with the differ lowercase letters in the same column for each analysis differ significantly (*p* < 0.05) Statistical significance: *p* < 0.001: highly significant; *p* < 0.01: very significant; *p* < 0.05: significant.

*Spirulina* is a rich source of gallic acid, caffeic acid, and p‐coumaric acid, which contribute to its antioxidant properties (Papalia et al. 2019). Therefore, adding *Spirulina* to kombucha increased the tea's existing phenolic content through its natural biochemical contributions.

Additionally, Kombucha fermentation involves microbial activities that can significantly transform and increase the concentrations of phenolic compounds. Specifically, beneficial bacteria and yeast species found in kombucha cultures have the ability to metabolize phenolic compounds and convert them into more complex derivatives (Singla et al. 2022). The presence of *Spirulina* can optimize fermentation conditions by increasing the bioavailability of phenolic acids through microbial interactions that inhibit oxidative degradation. This process helps maintain and even increase the levels of these compounds in the final product.

*H. pluvialis* has a high ability to produce astaxanthin, a potent antioxidant carotenoid. Astaxanthin synthesis occurs during the palmella stage, when *H. pluvialis* cells transition from active division to a phase focused on reserve accumulation, leading to an increase in astaxanthin levels (Rudi et al. 2025). As fermentation progresses, the microbial community containing both bacteria and yeast contributes to the breakdown of complex phenolics into simpler, more easily absorbed forms. This could explain the observed increases in bioactive compounds, including not only astaxanthin but also other polyphenols such as quercetin and kaempferol (Kitwetcharoen et al. 2023). Furthermore, the antioxidants produced during microbial metabolism can create a favorable environment for the growth of *H. pluvialis* and other beneficial effects, thereby promoting an increase in these compounds in the final beverage product.

Additionally, the fermentation process catalyzes the conversion of polyphenols in tea into more bioavailable forms, leading to an increase in their levels. The synergistic interactions of various bacteria and yeasts within the SCOBY facilitate the microbial conversion of plant‐based phenolics into bioactive metabolites (Doğan and Doğan 2023). Kombucha fermentation involves significant modification of the tea's own polyphenolic compounds, especially during long‐term fermentation periods when microbial enzymatic activities are increased (Ojo and Smidt 2023).

## 4 Conclusion

In this study, the effects of adding *S. maxima* and *H. pluvialis* to kombucha tea on the physicochemical and biological properties of kombucha teas were investigated.

Compared to *H. pluvialis*, the addition of *S. maxima* was found to be more effective in terms of the pH, pellicle weight, total antioxidant capacity, total phenolic content, textural values, microbiological development, and organic acid content of the teas. The addition of *H. pluvialis*, on the other hand, has been shown to add more value, especially in terms of brix, color, and some phenolic compounds such as astaxanthin, quercetin, and kaempferol in the samples. The addition of both microalgae had a much greater impact compared to the control samples, especially in terms of organic acid content, textural values, and phenolic profile.

The addition of *S. maxima* and *H. pluvialis* to kombucha production offers multifaceted benefits, including enhanced nutritional value, potent antioxidant properties, and enriched bioactive compounds. These features not only enhance the health benefits of kombucha but also position it as a more competitive functional beverage in the health food market.

As consumer awareness of health and healthy living continues to grow, the innovative incorporation of microalgae into traditional fermented beverages could signify a significant advancement in the field of functional food development.

As the food industry continues to seek innovative and health‐focused products today, the addition of such microalgae will not only meet consumer demand for functional foods but will also support sustainability in food production.

From an industrial perspective, the incorporation of *S. maxima* and *H. pluvialis* into kombucha presents a scalable opportunity to develop novel clean‐label functional beverages with enhanced bioactive profiles and naturally attractive coloration. However, the present study has several limitations. First, the bioactive properties were evaluated exclusively through in vitro analyses, which may not fully reflect their biological effects in vivo. Second, the relatively low number of biological replicates (two independent fermentation batches) may limit the statistical power of the two‐factor ANOVA and the generalizability of the findings. Future studies should therefore include a greater number of independent fermentation trials to improve the robustness and reliability of the statistical analyses. Furthermore, future research should focus on in vivo bioavailability studies, comprehensive sensory evaluations involving consumer panels, fermentation kinetics, titratable acidity measurements, and long‐term storage stability assessments to better validate the commercial potential and health benefits of microalgae‐fortified kombucha beverages.

## Author Contributions

**Gökhan Akarca:** writing – original draft, funding acquisition, project administration, supervision, resources, methodology. **Ayşe Janseli Denizkara:** writing – review and editing, methodology, formal analysis, software, data curation, investigation.

## Funding

The authors have nothing to report.

## Conflicts of Interest

The authors declare no conflicts of interest.

## Data Availability

The data that support the findings of this study are available from the corresponding author upon reasonable request.
